# Association of corticosteroids and other immunosuppressive medications with the severity and outcomes of diverticulitis: propensity score-matched retrospective study

**DOI:** 10.1093/bjsopen/zrag198

**Published:** 2026-11-13

**Authors:** Johannes M Salusjärvi, Laura Koskenvuo, Juha P Mali, Akseli Bonsdorff, Ari K Leppäniemi, Panu J Mentula, Ville J Sallinen

**Affiliations:** Gastroenterological Surgery, Helsinki University Hospital and University of Helsinki, Helsinki, Finland; Gastroenterological Surgery, Helsinki University Hospital and University of Helsinki, Helsinki, Finland; Gastroenterological Surgery, Helsinki University Hospital and University of Helsinki, Helsinki, Finland; Gastroenterological Surgery, Helsinki University Hospital and University of Helsinki, Helsinki, Finland; Department of surgery, Central Finland Central Hospital, Jyväskylä, Finland; Gastroenterological Surgery, Helsinki University Hospital and University of Helsinki, Helsinki, Finland; Gastroenterological Surgery, Helsinki University Hospital and University of Helsinki, Helsinki, Finland; Gastroenterological Surgery, Helsinki University Hospital and University of Helsinki, Helsinki, Finland; Transplantation and Liver Surgery, Helsinki University Hospital and University of Helsinki, Helsinki, Finland

## Abstract

**Background:**

Immunosuppressive medication (IM) is considered a risk factor for disease severity as well as outcomes in colonic diverticulitis. This study assessed the association of corticosteroid medications (CM) and other IMs separately on the severity and outcomes of acute diverticulitis.

**Methods:**

Patients admitted to Helsinki University Hospital due to verified acute colonic diverticulitis between 2006 and 2017 were included. Patients receiving CM were propensity score matched 1 : 1 to non-users (CM-control) with a similar risk profile for severe diverticulitis. Patients on other IMs were similarly matched to non-users (IM-control; 1 : 2).

**Results:**

Of 3219 diverticulitis episodes, 189 (5.9%) occurred during CM use and 121 (3.8%) during the use of other IM. There were 179 episodes of diverticulitis in both the CM and CM-control groups, 121 in the IM group, and 242 in the IM-control group. The rate of stage ≥ 3 diverticulitis was higher in CM than CM-control group (101 (56.4%) *versus* 57 (31.8%); *P* < 0.001). A higher corticosteroid dose was associated with higher rate of complicated diverticulitis (odds ratio 1.55 for 10-mg (prednisolone equivalent) increment in daily dose; 95% confidence interval 1.31 to 1.84). Other IMs were not associated with increased disease severity. Mortality and the need for intensive care were similar in CM and CM-control groups, as well as in the IM and IM-control groups, when analysed within the same diverticulitis stage.

**Conclusion:**

Corticosteroid use was dose-dependently associated with increasing severity of diverticulitis, whereas other IMs were not associated with increased disease severity. Neither CM nor other IM was associated with worse outcomes after stratification by diverticulitis stage.

## Teaser

From 3219 episodes of diverticulitis, 179 episodes in patients using corticosteroid medication (CM) were propensity score matched 1 : 1 to non-CM users with a similar risk profile for severe diverticulitis. CM was dose-dependently associated with higher diverticulitis stage, whereas no association was found between other immunosuppressive medications and severe diverticulitis. Neither CM nor other immunosuppressive medication was associated with outcomes within a diverticulitis stage.

## Introduction

Acute diverticulitis is a common reason for emergency medical assessment and can range from self-limiting uncomplicated disease to life-threatening peritonitis with organ dysfunction^1–3^. Because diverticulitis is a disease of infection and inflammation, medications that alter the immune and inflammatory systems may alter the course of the disease^4^. The increasing number of patients undergoing transplantations who require life-long immunosuppression, along with the rise of immunological therapies for autoimmune diseases, has led to an increase in the number of immunosuppressed patients^5^.

Current knowledge of the effects of immunosuppressive medications on the course and outcomes of acute diverticulitis is limited. Antibiotic treatment offers little, if any, benefit in the treatment of uncomplicated diverticulitis^6–8^. Nevertheless, clinical practice guidelines recommend antibiotic treatment for patients on immunosuppressive medication^9,10^, because non-antibiotic treatment has not been studied in this patient group. Several studies^11–18^ have reported that immunosuppressed patients are at increased risk of diverticulitis complications such as abscess or peritonitis. A recent meta-analysis^19^ reported that patients treated with immunosuppressive medication are also at almost double the risk of mortality after surgery for acute diverticulitis. However, most studies do not distinguish between corticosteroid medication (CM) and other types of immunosuppression, and do not consider different doses of corticosteroids^11–17^. Further, many studies use a binary classification of diverticulitis (that is, uncomplicated or complicated)^14–16,18^. Complicated diverticulitis includes a wide range of stages, from small abscesses with near zero mortality to peritonitis and organ failure with over 30% mortality^1^. It is currently unclear whether all immunosuppressive medications affect the severity and outcomes of diverticulitis in the same way.

The aim of this study was to assess whether CM and other immunosuppressive medications were associated with the stage (severity) of diverticulitis or the outcomes of treatment within these stages.

## Methods

This was a single-centre retrospective cohort study performed in Helsinki University Hospital (HUH). HUH serves as a secondary (for a population of 0.7 million) and tertiary (for a population of 1.7 million) referral centre and is an academic teaching hospital. Patient records of all patients admitted to HUH between 1 January 2006 and 31 December 2017 with International Classification of Diseases, Tenth Revision (ICD-10) diagnosis codes K57 (diverticular disease of the intestine), K63.0 (abscess of intestine), K63.1 (perforation of intestine), and K63.2 (fistula of intestine) were manually screened. Patients with colonic diverticulitis verified either by a radiologist after computed tomography (CT) or by a surgeon after emergency surgery were included. Patients whose disease proved to be other than diverticulitis (after pathology report, surgery, or endoscopy) were excluded. Diverticulitis episodes with only a clinical diagnosis were not included in the study.

Diagnostic and treatment protocols underwent some changes during the study period. In 2006, CT imaging was used at the discretion of the on-call surgeon, but this has shifted to more routine use of CT imaging, whereby patients generally undergo CT verification for their first episodes of diverticulitis or if complicated diverticulitis is suspected. With regard to treatment, until 2014, uncomplicated diverticulitis was routinely treated with antibiotics. During the period 2014–2015, patients with uncomplicated diverticulitis were recruited to a single-arm trial on symptomatic treatment without antibiotics^6^ and, after the trial, from 2016, a no-antibiotic policy has been followed for uncomplicated diverticulitis unless antibiotic treatment is warranted by, for example, immunosuppression. Operative treatment is chosen at the discretion of the on-call surgeon, generally if a patient has peritonitis, bowel obstruction, or the disease has not responded to conservative treatment. The use of laparoscopic lavage as the only treatment is not encouraged at the HUH.

The severity of each individual episode of diverticulitis was assessed in the present study using the Helsinki 2.0 staging for acute diverticulitis^20^, as follows: stage 1, uncomplicated diverticulitis (pericolic gas allowed); stage 2, complicated diverticulitis with small abscess (< 5 cm); stage 3, complicated diverticulitis with large abscess (≥ 5 cm) or distant intraperitoneal or retroperitoneal air (distant gas defined as gas found > 50 mm from the inflamed bowel segment); stage 4, generalized peritonitis without organ dysfunction; and stage 5, generalized peritonitis with organ dysfunction, or faecal peritonitis during surgery. Stage 3 was defined as the cut-off for severe diverticulitis, because the need for invasive treatment is markedly higher with stages ≥ 3 than for stages 1 and 2^20^. The modified Hinchey classification^21^ was used alongside the Helsinki 2.0 staging for comparison, and was defined as follows: 1a, phlegmon; 1b, pericolic abscess; 2, pelvic abscesses; 3, purulent peritonitis; 4, faecal peritonitis^21^. Readmissions within 30 days were considered as a continuation of the index episode. If more than 30 days had passed since the previous discharge, the next admission was analysed as a new episode. Long-term illnesses were assessed using Charlson co-morbidity index (CCI). Daily corticosteroid dose at the time of admission was transformed to equivalent prednisolone dose according to the Oxford University *Handbook of Critical Care*^22^ dose algorithm to allow comparison between different corticosteroid drugs. Mortality is reported as 30-day mortality from admission.

Patients on immunosuppressants (especially those on CM) are not comparable to other diverticulitis patients in terms of age and co-morbidities. Thus, propensity score (PS) matching was used to control for confounders. The PS was calculated using logistic regression analysis and matching was performed in a descending nearest-neighbour manner with an accepted calliper of < 0.25. The matching ratio was set at 1 : 1 or 2 : 1, if feasible, and the matched cohort balance was assessed using standardized mean differences, whereby values < 0.1 indicated an adequate balance for covariates. Variables that were assumed to be associated with a higher stage of diverticulitis and outcomes, as per the directed acyclic graph in *Fig. S1*, were selected as covariates for PS matching: age, need for outside assistance, sex, diabetes, chronic kidney disease, chronic liver disease, chronic obstructive pulmonary disease, asthma, dementia, peripheral vascular disease, history of cerebrovascular infarction, history of myocardial infarction, congestive heart failure, cytotoxic medication, and history of previous diverticulitis. Patients were divided into two groups according to their use of immunosuppressants at the time of admission to hospital: CM and immunosuppressive medications other than corticosteroids (IM group). Patients on both CM and immunosuppressive medications other than corticosteroids were included in both groups. PS matching was used to match patients in the CM and IM groups with respective control groups (CM-control and IM-control, respectively). The CM-control comprised patients not using CM; similarly, the IM-control group comprised patients not using immunosuppressive medication other than corticosteroids. The use of immunosuppressive medication other than corticosteroids was used as a matching variable between the CM and CM-control groups, and corticosteroid use was used as a matching variable between the IM and IM-control groups.

Continuous variables with a skewed distribution are presented as the median and interquartile range (i.q.r.) and were compared using the Mann–Whitney *U* test. Categorical variables were compared using χ^2^ tests. Binary logistic regression was used for effect size analysis. Two-tailed *P* < 0.05 was considered significant. Data were analysed using SPSS^®^ version 28 (IBM, Armonk, NY, USA). PS matching was performed using the MatchIt package in R.

This study was approved by the HUH Institutional Review Board.

## Results

The study cohort included 3219 episodes of verified acute diverticulitis in 2860 unique patients. CM was used at the time of admission in 189 episodes (5.9%), and other immunosuppressive medications were used in 121 episodes (3.8%). Baseline demographics for all patients and patients using CM, other immunosuppressive medications, and no immunosuppressive medications are presented in *Table 1*. Sixty-three patients were using both CM and other immunosuppressive medication. Compared with patients not using any immunosuppressive medication, patients in the CM and IM groups were older (58.1 *versus* 70.7 and 65.6 years, respectively), fewer lived without outside assistance (96.9% *versus* 83.1% and 92.6%, respectively), were more often female (64.3% *versus* 74.1% and 76.0%, respectively), had more co-morbidities (median CCI 0 *versus* 2 and 1, respectively), and were more often on cytotoxic medication (39 (1.3%) *versus* 14 (7.4%) and 3 (2.5%), respectively). In all, 2311 diverticulitis episodes (77.8%) were uncomplicated in patients not taking any immunosuppressive medication, compared with 61 (32.3%) and 73 (60.3%) diverticulitis episodes in the CM and IM groups, respectively. In patients not taking any immunosuppressive medication, 170 (5.7%) episodes of diverticulitis were complicated with generalized peritonitis (Helsinki 2.0 stage 4 or 5), compared with 69 (36.5%) and 23 (19.0%) episodes in the CM and IM groups, respectively (*Table 1*).

**Table 1:** Patient demographics overall and according to CM and other IM status

	All patients	Patients without CM or other IM	Patients using other IM	Patients using CM
No. of patients*	3219	2972	121	189
No. of unique patients*	2860	2647	94	167
Patients with > 1 episode	287 (10.0%)	261 (9.8%)	13 (14%)	19 (11.4%)
Age (years), median (i.q.r.)	59.1 (49.9–69.2)	58.1 (49.2–68.2)	65.6 (58.0–74.0)	70.7 (63,2–78.2)
**Need for assistance before admission**				
No need	3092 (96.1%)	2879 (96.9%)	112 (92.6%)	157 (83.1%)
Home care	63 (2.0%)	47 (1.6%)	4 (3.3%)	14 (7.4%)
Nursing home/ward	63 (2.0%)	46 (1.5%)	5 (4.1%)	18 (9.5%)
**Sex**				
Female	2091 (65.0%)	1912 (64.3%)	92 (76.0%)	140 (74.1%)
Male	1128 (35.0%)	1060 (35.7%)	29 (24.0%)	49 (25.9%)
Charlson co-morbidity index, median (i.q.r.)	0 (0–1)	0 (0–1)	1 (1–3)	2 (1–4)
Diabetes	332 (10.3%)	290 (9.8%)	17 (14.0%)	35 (18.5%)
CKD stage 4–5†	39 (1.2%)	18 (0.6%)	13 (10.7%)	17 (9.0%)
**Liver disease**				
Mild	15 (0.5%)	14 (0.5%)	1 (0.8%)	0
Moderate/severe	5 (0.2%)	3 (0.1%)	2 (1.7%)	1 (0.5%)
COPD	92 (2.9%)	74 (2.5%)	4 (3.3%)	16 (8.5%)
Asthma	345 (10.7%)	289 (9.7%)	22 (18.2%)	48 (25.4%)
Dementia	71 (2.2%)	62 (2.1%)	2 (1.7%)	9 (4.8%)
Peripheral vascular disease	69 (2.1%)	54 (1.8%)	7 (5.8%)	12 (6.3%)
History of a cerebrovascular infarction	46 (1.4%)	40 (1.3%)	3 (2.5%)	4 (2.1%)
History of myocardial infarction	119 (3.7%)	97 (3.3%)	8 (6.6%)	18 (9.5%)
Congestive heart failure	91 (2.8%)	63 (2.1%)	10 (8.3%)	26 (13.8%)
Corticosteroid medication	189 (5.9%)	0	63 (52.1%)	189 (100%)
Cytotoxic medication or ongoing chemotherapy	54 (1.7%)	39 (1.3%)	3 (2.5%)	14 (7.4%)
**Other immunosuppressive medication**	121 (3.8%)	0	121 (100%)	63 (33.3%)
Methotrexate	61 (1.9%)		61 (50.4%)	36 (19.0%)
Hydroxychloroquine	23 (0.7%)		23 (19.0%)	14 (7.4%)
Cyclosporine	14 (0.4%)		14 (11.6%)	8 (4.2%)
Azathioprine	11 (0.3%)		11 (9.1%)	1 (0.5%)
Sulfasalazine	11 (0.3%)		11 (9.1%)	2 (1.1%)
Mycophenolate	9 (0.3%)		9 (7.4%)	4 (2.1%)
Tacrolimus	8 (0.2%)		8 (6.6%)	2 (1.1%)
TNF-α inhibitors	7 (0.2%)		7 (5.8%)	3 (1.6%)
Other‡	20 (0.6%)		20 (16.5%)	13 (6.9%)
First diagnosed episode of diverticulitis	2404 (74.7%)	2220 (74.7%)	83 (68.6%)	146 (77.2%)
**Helsinki stage 2.0**				
1	2420 (75.2%)	2311 (77.8%)	73 (60.3%)	61 (32.3%)
2	314 (9.8%)	291 (9.8%)	14 (11.6%)	19 (10.1%)
3	213 (6.6%)	175 (5.9%)	9 (7.4%)	37 (19.6%)
4	140 (4.3%)	106 (3.6%)	13 (10.7%)	31 (16.4%)
5	104 (3.2%)	64 (2.2%)	10 (8.3%)	38 (20.1%)
Obstruction/fistula	28 (0.9%)	25 (0.8%)	2 (1.7%)	3 (1.6%)
**Modified Hinchey classification**				
1a	2483 (77.1%)	2363 (79.5%)	75 (62.0%)	71 (37.6%)
1b	333 (10.3%)	302 (10.2%)	15 (12.4%)	29 (15.3%)
2	132 (4.1%)	113 (3.8%)	6 (5.0%)	17 (9.0%)
3	176 (5.5%)	130 (4.4%)	18 (14.9%)	42 (22.2%)
4	61 (1.9%)	35 (1.2%)	4 (3.3%)	25 (13.2%)
Unclassified peritonitis	6 (0.2%)	4 (0.1%)	1 (0.8%)	2 (1.1%)
Obstruction/fistula	28 (0.9%)	25 (0.8%)	2 (1.7%)	3 (1.6%)
**Primary treatment**				
Conservative	2875 (89.3%)	2705 (91.0%)	96 (79.3%)	112 (59.3%)
No antibiotics/antibiotics	673/2202	660/2045	10/111	5/107
Drainage	57 (1.8%)	53 (1.8%)	1 (0.8%)	4 (2.1%)
Surgery	287 (8.9%)	214 (7.2%)	24 (19.8%)	73 (38.6%)
Diverticulitis progresses (stage increases) during treatment	27 (0.8%)	22 (0.7%)	3 (2.5%)	2 (1.1%)
**Bowel resection as primary treatment**	272 (8.4%)	198 (6.7%)	23 (19.0%)	70 (37.0%)
Anastomosis	102 (37.5%)	90 (45.5%)	6 (26%)	11 (16%)
Anastomotic leak	4 (3.9%)	2 (2%)	1 (17%)	2 (18%)
LOS (days), median (i.q.r.)	1 (0–3)	1 (0–3)	3 (1–8)	7 (2–12)
Mortality within 30 days of admission	59 (1.8%)	28 (0.9%)	7 (5.8%)	31 (16.4%)

Values are *n* (%) unless otherwise stated. Helsinki 2.0 staging was as follows: stage 1, uncomplicated diverticulitis (pericolic gas allowed); stage 2, complicated diverticulitis with small abscess (< 5 cm); stage 3, complicated diverticulitis with large abscess (≥ 5 cm) or distant intraperitoneal or retroperitoneal air; stage 4, generalized peritonitis without organ dysfunction; stage 5, generalized peritonitis with organ dysfunction, or faecal peritonitis during surgery. The modified Hinchey classification was as follows: 1a, phlegmon; 1b, pericolic abscess; 2, pelvic abscess; 3, purulent peritonitis; 4, faecal peritonitis. *There were 3219 diverticulitis episodes from 2860 unique patients. †CKD stage 4–5 was defined as a glomerular filtration rate < 30 ml/min/1.73 m^2^, dialysis, or kidney transplant. ‡Other includes four patients on leflunomide, three patients on rituximab, three patients on abatacept, two patients on anakinra, and one patient on each of the following: intravenous immunoglobulin, colchicine, cyclophosphamide, lenalidomide, bevacizumab, glatiramer acetate, sirolimus, hydroxycarbamide and ustekinumab. CM, corticosteroid medication; IM, immunosuppressive medication (other than corticosteroids); i.q.r., interquartile range; CKD, chronic kidney disease; COPD, chronic obstructive pulmonary disease; TNF-α, tumour necrosis factor-α; LOS, length of hospital stay.

As indicated in *Table 1*, 660 (22.2%), 5 (2.6%), and 10 (8.3%) patients in the no immunosuppressive medication, CM, and IM groups, respectively, received conservative treatment without antibiotics as the primary treatment. Bowel resection as the primary treatment was performed in 198 (6.7%), 70 (37.0%) and 23 (19.0%) patients in the no immunosuppressive medication, CM, and IM groups, respectively. Laparoscopic lavage as the primary treatment was performed in six patients, one of whom was on both CM and other immunosuppressive medication. Mortality within 30 days of admission was 0.9% (28 patients) among patients not on any immunosuppressive medication, compared with 16.4% (31 patients) and 5.8% (7 patients) in the CM and IM groups, respectively. Over twice as many patients were included in the study for the period 2012–2017 compared with 2006–2011 (2262 *versus* 957, respectively; *Table S1*). Most of this increase in patient numbers was due to patients with uncomplicated diverticulitis (*Table S1*). There were no significant differences between the period 2006–2011 and 2012–2017 in the percentage of patients on CM (65 (6.8%) *versus* 124 (5.5%), respectively; *P* = 0.173) or other immunosuppressive medications (34 (3.6%) *versus* 87 (3.8%), respectively; *P* = 0.765).

Patients using corticosteroids were matched 1 : 1 with patients not using CM because not enough suitable control patients could be identified during PS matching using a ratio of 1 : 2. Ten patients using corticosteroids were not matched and were subsequently excluded because no suitable match within the specified calliper width could be found. Patients using other immunosuppressive medications were matched 1 : 2 with patients not using immunosuppressive medication other than corticosteroids. The standardized mean differences between the matched CM and CM-control groups, as well as between the matched IM and IM-control groups, were < 0.2, with most even < 0.1 (*Tables S2* and *S3*).

The demographics of the matched cohorts are presented in *Table 2*. There were no differences between the CM and CM-control groups except that patients in the CM-control group were a median of 2.1 years older (*P* = 0.038) and had CCI scores that were a median of 1 point lower (*P* < 0.001). There were no differences between the IM and IM-control groups in any of the variables. The two most common indications for CM were various connective tissue diseases (CTDs; 85 patients, 47.5%) and tumour/malignancy (40 patients, 22.3%; *Table S4*). The median daily corticosteroid dose was markedly higher in patients with tumour/malignancy (60 mg prednisolone equivalent) than in patients with other indications (5–16.3 mg; *Table S4*). For immunosuppressive medication other than corticosteroids, the two most common indications were CTDs (78 patients, 64.5%) and organ transplant (16 patients, 13.2%; *Table S5*).

**Table 2:** Demographics of patients on CM or other IM and their respective propensity score-matched control groups

	CM	CM-control	P*	IM	IM-control	P*
No. of patients	179	179		121	242	
Age (years), median (i.q.r.)	70.5 (62.7–77.8)	72.6 (64.1–82.7)	0.038†	65.6 (58.0–74.0)	69.0 (57.9–77.6)	0.121†
**Need for assistance before admission**			0.672			0.910
No need	148 (82.7%)	147 (82.1%)		112 (92.6%)	221 (91.3%)	
Home care	14 (7.8%)	18 (10.1%)		4 (3.3%)	10 (4.1%)	
Nursing home/ward	17 (9.5%)	14 (7.8%)		5 (4.1%)	11 (4.5%)	
**Sex**			0.634			1.000
Female	133 (74.3%)	128 (71.5%)		92 (76.0%)	184 (76.0%)	
Male	46 (25.7%)	51 (28.5%)		29 (24.0%)	58 (24.0%)	
Charlson co-morbidity index, median (i.q.r.)	2 (1–4)	1 (0–2)	<0.001†	1 (1–3)	1 (0–3)	0.371†
Diabetes	34 (19.0%)	38 (21.2%)	0.692	17 (14.0%)	41 (16.9%)	0.577
CKD stage 4–5‡	11 (6.1%)	8 (4.5%)	0.637	13 (10.7%)	16 (6.6%)	0.245
**Liver disease**			0.220			0.837
Mild	0	2 (1.1%)		1 (0.8%)	1 (0.4%)	
Moderate/severe	0	1 (0.6%)		2 (1.7%)	3 (1.2%)	
COPD	16 (8.9%)	18 (10.1%)	0.857	4 (3.3%)	13 (5.4%)	0.539
Asthma	47 (26.3%)	43 (24.0%)	0.715	22 (18.2%)	52 (21.5%)	0.549
Dementia	8 (4.5%)	9 (5.0%)	1.000	2 (1.7%)	6 (2.5%)	0.899
Peripheral vascular disease	10 (5.6%)	9 (5.0%)	1.000	7 (5.8%)	9 (3.7%)	0.527
History of a cerebrovascular infarction	4 (2.2%)	2 (1.1%)	0.681	3 (2.5%)	5 (2.1%)	1.000
History of myocardial infarction	17 (9.5%)	18 (10.1%)	1.000	8 (6.6%)	19 (7.9%)	0.832
Congestive heart failure	24 (13.4%)	24 (13.4%)	1.000	10 (8.3%)	25 (10.3%)	0.660
Cytotoxic medication or ongoing chemotherapy	13 (7.3%)	9 (5.0%)	0.509	3 (2.5%)	9 (3.7%)	0.756
Corticosteroid medication	179 (100%)	0		63 (52.1%)	121 (50.0%)	0.795
**Other IM**	54 (30.2%)	47 (26.3%)	0.481	121 (100%)	0	
Methotrexate	33 (18.4%)	19 (10.6%)	0.051	61 (50.4%)		
Hydroxychloroquine	13 (7.3%)	8 (4.5%)	0.368	23 (19.0%)		
Cyclosporine	5 (2.8%)	5 (2.8%)	1.000	14 (11.6%)		
Azathioprine	1 (0.6%)	9 (5.0%)	0.025	11 (9.1%)		
Sulfasalazine	2 (1.1%)	7 (3.9%)	0.177	11 (9.1%)		
Mycophenolate	2 (1.1%)	4 (2.2%)	0.681	9 (7.4%)		
Tacrolimus	1 (0.6%)	6 (3.4%)	0.127	8 (6.6%)		
TNF-α inhibitor	3 (1.7%)	3 (1.7%)	1.000	7 (5.8%)		
Other§	12 (6.7%)	5 (2.8%)	0.136	20 (16.5%)		
First diagnosed episode of diverticulitis	137 (76.5%)	139 (77.7%)	0.900	83 (68.6%)	180 (74.4%)	0.299
**Helsinki 2.0 stage**			<0.001			0.093
1	57 (31.8%)	105 (58.7%)		73 (60.3%)	134 (55.4%)	
2	18 (10.1%)	14 (7.8%)		14 (11.6%)	14 (5.8%)	
3	34 (19.0%)	23 (12.8%)		9 (7.4%)	36 (14.9%)	
4	29 (16.2%)	15 (8.4%)		13 (10.7%)	25 (10.3%)	
5	38 (21.2%)	19 (10.6%)		10 (8.3%)	31 (12.8%)	
Obstruction/fistula	3 (1.7%)	3 (1.7%)		2 (1.7%)	2 (0.8%)	
**Modified Hinchey classification**			<0.001			0.363
1a	67 (37.4%)	111 (62.0%)		75 (62.0%)	143 (59.1%)	
1b	26 (14.5%)	18 (10.1%)		15 (12.4%)	22 (9.1%)	
2	16 (8.9%)	13 (7.3%)		6 (5.0%)	19 (7.9%)	
3	40 (22.3%)	25 (14.0%)		18 (14.9%)	33 (13.6%)	
4	25 (14.0%)	7 (3.9%)		4 (3.3%)	22 (9.1%)	
Unclassified peritonitis	2 (1.1%)	2 (1.1%)		1 (0.8%)	1 (0.4%)	
Obstruction/fistula	3 (1.7%)	3 (1.7%)		2 (1.7%)	2 (0.8%)	

Values are *n* (%) unless otherwise stated. Helsinki 2.0 staging was as follows: stage 1, uncomplicated diverticulitis (pericolic gas allowed); stage 2, complicated diverticulitis with small abscess (< 5 cm); stage 3, complicated diverticulitis with large abscess (≥ 5 cm) or distant intraperitoneal or retroperitoneal air; stage 4, generalized peritonitis without organ dysfunction; stage 5, generalized peritonitis with organ dysfunction, or faecal peritonitis during surgery. The modified Hinchey classification was as follows: 1a, phlegmon; 1b, pericolic abscess; 2, pelvic abscess; 3, purulent peritonitis; 4, faecal peritonitis. ‡CKD stage 4–5 was defined as a glomerular filtration rate < 30 ml/min/1.73 m^2^, dialysis, or kidney transplant. §Other includes four patients on leflunomide, three patients on rituximab, three patients on abatacept, two patients on anakinra, and one patient on each of the following: intravenous immunoglobulin, colchicine, cyclophosphamide, lenalidomide, bevacizumab, glatiramer acetate, sirolimus, hydroxycarbamide, and ustekinumab. CM, corticosteroid medication; IM, immunosuppressive medication (other than corticosteroids); i.q.r., interquartile range; CKD, chronic kidney disease; COPD, chronic obstructive pulmonary disease; TNF-α, tumour necrosis factor-α. *Pearson χ^2^ for categorical variables (continuity correction for 2 × 2 variables) and †Mann–Whitney *U* test for continuous variables.

Complicated diverticulitis (Helsinki 2.0 stage 2 or higher) was more common in the CM than CM-control group (122 (68.2%) *versus* 74 (41.3%); *P* < 0.001), but there was no significant difference in the incidence of complicated diverticulitis between the IM and IM-control groups (48 (39.7%) *versus* 108 (44.6%), respectively; *P* = 0.354). The incidence of complicated diverticulitis increased with corticosteroid dose, and the odds ratio for complicated diverticulitis was 1.55 (95% confidence interval 1.31 to 1.84; *P* < 0.001) for every 10-mg (prednisolone equivalent) increment in daily corticosteroid dose (*Fig. 1*). Bowel resection was the primary treatment for 67 (37.4%) patients in the CM group and 39 (21.8%) patients in the CM-control group. Resection without an anastomosis was performed in 57 (85%) and 27 (69%) patients in the CM and CM-control groups, respectively (*P* = 0.091). In the IM and IM-control groups 23 (19.0%) and 55 (22.7%) patients, respectively, underwent bowel resection as the primary treatment. Resection without an anastomosis was performed in 17 (74%) and 47 (86%) patients in the IM and IM-control groups, respectively (*P* = 0.375). The rate of severe diverticulitis (Helsinki 2.0 stage ≥ 3) was higher in the CM than CM-control group (101 (56.4%) *versus* 57 (31.8%); *P* < 0.001). The incidence of severe diverticulitis increased with corticosteroid dose and, among the 49 patients with corticosteroid doses exceeding 20 mg/day (prednisolone equivalent), 44 had severe diverticulitis (*Fig. 1* and *Table S6*). The odds ratio for severe diverticulitis was 1.63 (95% confidence interval 1.38 to 1.91; *P* < 0.001) for every 10-mg (prednisolone equivalent) increment in daily corticosteroid dose. Other immunosuppressive medications were not associated with an increased rate of severe diverticulitis (26.4% *versus* 38.0% in the IM and IM-control groups, respectively; *P* = 0.043)

**Figure 1:**
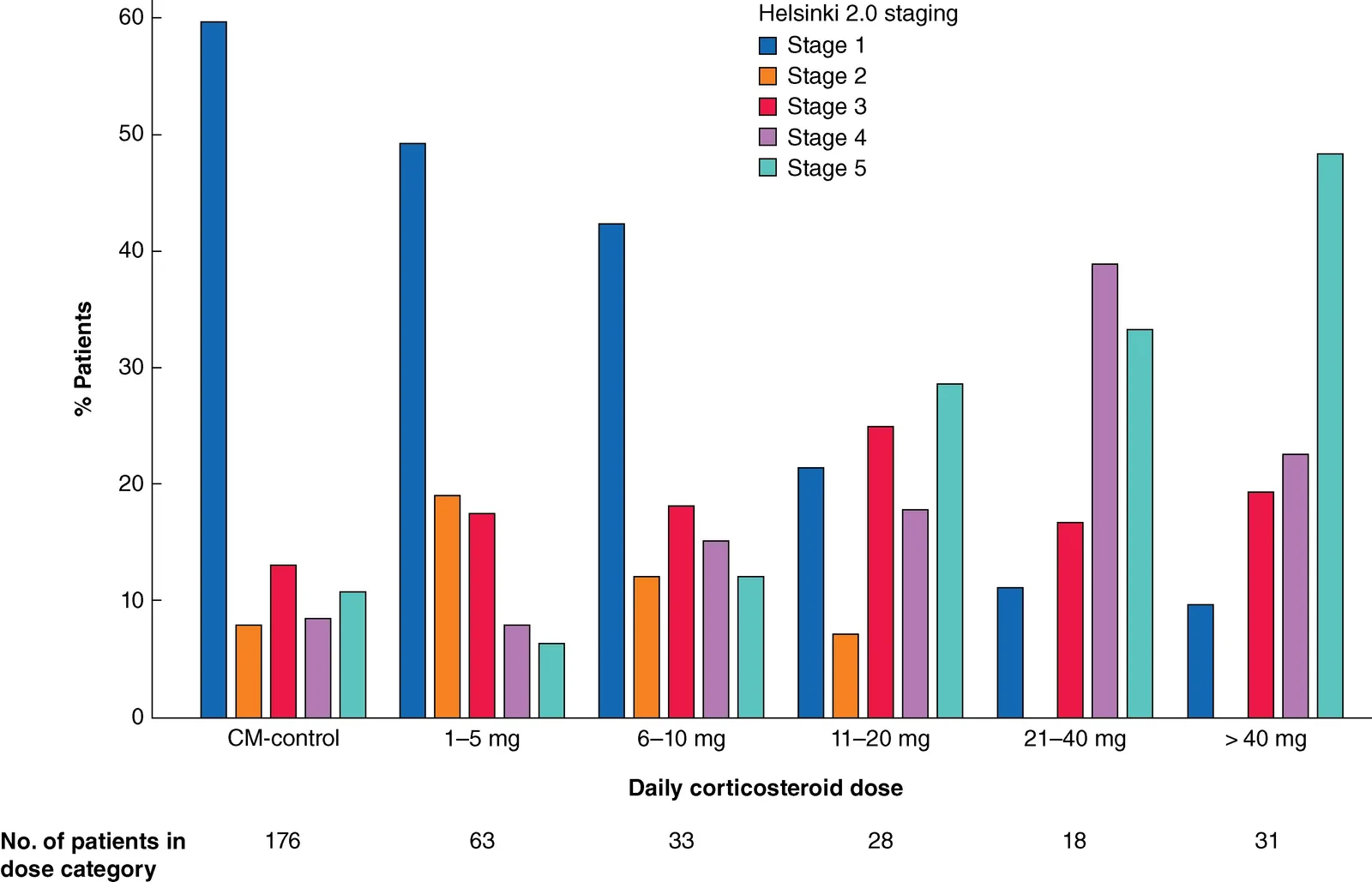
Severity of diverticulitis according to daily corticosteroid dose (prednisolone equivalent) in the CM group and its propensity score-matched control Diverticulitis stage distribution changed with corticosteroid dose, with the percentage of patients with higher stages increasing with corticosteroid dose. Helsinki 2.0 staging was as follows: stage 1, uncomplicated diverticulitis (pericolic gas allowed); stage 2, complicated diverticulitis with small abscess (< 5 cm); stage 3, complicated diverticulitis with large abscess (≥ 5 cm) or distant intraperitoneal or retroperitoneal air; stage 4, generalized peritonitis without organ dysfunction; stage 5, generalized peritonitis with organ dysfunction, or faecal peritonitis during surgery. CM, corticosteroid medication.

Because CTDs could not be used as a confounder in PS matching (there were not enough control patients with CTD but no CM), a sensitivity analysis was performed by excluding patients with CTD in the CM and CM-control groups (99 (55.3%) and 22 (12.3%) patients, respectively) from the analysis. The rate of complicated diverticulitis in this sensitivity analysis (excluding patients with CTD) was still higher in the CM than CM-control group (74% (59) *versus* 42.7% (67); *P* < 0.001). A similar analysis excluding CTD patients from the IM and IM-control groups was performed (78 (64.5%) and 54 (22.3%) patients excluded, respectively), and no difference was found in the rate of complicated diverticulitis (26% (11) and 39.4% (74), respectively; *P* = 0.130). When patients with metastatic malignancy and/or ongoing treatment for cancer were excluded from the CM and CM-control groups (42 (23.5%) and 9 (5.0%) patients, respectively), the rate of complicated diverticulitis remained higher in the CM than CM-control group (59.9% (82) *versus* 40.0% (68); *P* < 0.001).

In CM and CM-control groups, 13 (8.2%) and 6 (3.5%) unique patients, respectively, had two or more episodes of diverticulitis included. In the IM and IM-control groups, 13 (13.0%) and 8 (3.5%) unique patients, respectively, had two or more episodes of diverticulitis included. A sensitivity analysis was performed by including only the first diverticulitis episode from each unique patient and excluding the later episodes from the cohorts (20, 7, 21, and 11 episodes excluded from the CM, CM-control, IM, and IM-control groups, respectively). The rate of complicated diverticulitis remained higher in the CM than CM-control group (69.2% (110) *versus* 41.9% (72); *P* < 0.001), but no difference was found between the IM and IM-control groups (42.0% (42) *versus* 43.7% (101), respectively; *P* = 0.865).

Overall, 30-day mortality was higher in the CM than CM-control group (29 (16.5%) *versus* 12 (6.8%); *P* = 0.008; *Table 3*). In addition, in the CM *versus* CM-group, the median length of hospital stay was longer (6.5 *versus* 2 days; *P* < 0.001), more patients required surgical intervention (82 (46.6%) *versus* 50 (28.4%); *P* < 0.001), more patients required intensive care (24 (13.6%) *versus* 9 (5.1%); *P* = 0.010), and the mean intensive care-free days was lower (26 *versus* 28; *P* < 0.001). When outcomes were compared within different stages of diverticulitis specifically, no significant differences were noted between the CM and CM-control groups, except for a longer median length of hospital stay in the CM group for uncomplicated diverticulitis (1 (i.q.r. 0–3) *versus* 1 (i.q.r. 0–2) days, respectively; *P* = 0.008; *Table 3*). Similarly, for uncomplicated diverticulitis, the IM group had a longer median length of hospital stay than the IM-control group (1 (i.q.r. 0–3) *versus* 0 (i.q.r. 0–2) days, respectively; *P* = 0.015); all other outcomes were similar between the IM and IM-control groups in the whole matched cohort as well as within different diverticulitis stages (*Table 3*). Similar results were obtained when patients were stratified using the modified Hinchey-classification (*Table S7*).

**Table 3:** Stage-specific outcomes for patients on CM or other IM and their respective propensity score-matched control groups

	Helsinki 2.0 stage‡	CM	CM-control	P*	IM	IM-control	P*
30-day mortality	1	1 (2%)	1 (1.0%)	1.000	0 (0%)	2 (1.5%)	0.760
	2	3 (17%)	0 (0%)	0.321	1 (7%)	2 (14%)	1.000
	3	3 (9%)	1 (4%)	0.904	1 (11%)	4 (11%)	1.000
	4	6 (21%)	2 (13%)	0.851	1 (8%)	5 (20%)	0.604
	5	16 (42%)	8 (42%)	1.000	3 (30%)	12 (39%)	0.905
	All stages	29 (16.5%)	12 (6.8%)	0.008	6 (5.0%)	25 (10.4%)	0.132
LOS (days), median (i.q.r.)	1	1 (0–3)	1 (0–2)	0.008†	1 (0–3)	0 (0–2)	0.015†
	2	4.5 (3–6)	4 (1–8)	0.722†	3.5 (2–5)	4.5 (3–6)	0.511†
	3	8 (7–14)	9 (4–15)	0.845†	14 (8–21)	8 (6–11)	0.232†
	4	9 (7–13)	10.0 (7–13)	0.881†	10 (8–18)	10 (8–13)	0.447†
	5	13 (7–19)	7 (4–20)	0.278†	16 (11–22)	12 (8–19)	0.463†
	All stages	6.5 (2–12)	2 (0–7)	< 0.001†	3 (1–8)	3 (0–9)	0.655†
Surgical intervention during treatment period	1	1 (2%)	1 (1.0%)	1.000	1 (1%)	1 (0.7%)	1.000
	2	1 (6%)	4 (29%)	0.198	2 (14%)	2 (14%)	1.000
	3	15 (44%)	13 (57%)	0.516	5 (56%)	14 (39%)	0.597
	4	29 (100%)	15 (100%)		13 (100%)	25 (100%)	
	5	36 (95%)	17 (90%)	0.855	9 (90%)	30 (97%)	0.984
	All stages	82 (46.6%)	50 (28.4%)	< 0.001	30 (25.2%)	72 (30.0%)	0.411
Need for ICU during treatment period	1	1 (2%)	0 (0%)	0.756	1 (1%)	1 (0.7%)	1.000
	2	1 (6%)	1 (7%)	1.000	1 (7%)	1 (7%)	1.000
	3	4 (12%)	1 (4%)	0.621	2 (22%)	3 (8%)	0.553
	4	6 (21%)	2 (13%)	0.851	4 (31%)	3 (12%)	0.330
	5	12 (32%)	5 (26%)	0.918	5 (50%)	12 (39%)	0.794
	All stages	24 (13.6%)	9 (5%)	0.010	13 (10.9%)	20 (8%)	0.545
ICU-free days within 30 days, mean(s.d.)	1	30(2)	30(2)	0.257†	30(0)	30(2)	0.658†
	2	28(7)	30(0)	0.442†	28(8)	29(2)	0.804†
	3	28(5)	29(4)	0.353†	27(7)	28(5)	0.791†
	4	24(11)	27(6)	0.326†	26(9)	25(10)	0.716†
	5	20(11)	20(13)	0.814†	22(11)	21(11)	0.940†
	All stages	26(8)	28(6)	< 0.001†	28(6)	28(6)	0.462†

Values are *n* (%) unless otherwise stated. ‡Helsinki 2.0 staging was as follows: STAGE 1, uncomplicated diverticulitis (pericolic gas allowed); stage 2, complicated diverticulitis with small abscess (< 5 cm); stage 3, complicated diverticulitis with large abscess (≥ 5 cm) or distant intraperitoneal or retroperitoneal air; stage 4, generalized peritonitis without organ dysfunction; stage 5, generalized peritonitis with organ dysfunction, or faecal peritonitis during surgery. Helsinki 2.0 staging was not applicable to six patients in CM cohort and four patients in the other IM cohort (bowel obstruction). CM, corticosteroid medication; IM, immunosuppressive medication (other than corticosteroids); LOS, length of hospital stay; i.q.r., interquartile range; ICU, intensive care unit; s.d., standard deviation. *Pearson’s χ^2^ for categorical variables (continuity correction for 2 × 2 variables) and †Mann–Whitney *U* test for continuous variables.

Neither CM nor other IM was associated with disease progression (stage increase) during the hospital stay or within 30 days after discharge, with disease progression noted in 2 (1.1%) *versus* 2 (1.1%) patients in the CM and CM-control groups, respectively (*P* = 1.000), and in 3 (2.5%) *versus* 4 (1.7%) patients in the IM and IM-control groups, respectively (*P* = 0.893).

## Discussion

In this study, patients who were on CM had more severe diverticulitis, higher mortality, and required surgical interventions and intensive care unit stay more often than patients without CM. However, when these outcomes were assessed within different diverticulitis severity stages, no differences were noted between patients on CM and their matched controls. This suggests that the association between corticosteroid use and worse treatment outcomes may reflect greater disease severity at presentation, rather than differences in outcomes within severity strata. This was further supported by the finding that disease progression to a higher severity stage was similar (and rare) regardless of CM. The association between diverticulitis severity and CM was found to be dose dependent; that is, higher doses of corticosteroids were associated with more severe disease. In contrast, no similar associations were noted between other IMs and either diverticulitis severity stage or treatment outcomes, suggesting that the mechanisms behind the association of CM with more severe diverticulitis may be related to corticosteroid properties other than immunosuppression.

Previous research has predominantly addressed CM and the effects it has on the severity and outcomes of diverticulitis from the viewpoint of immunosuppression. In these studies^12,13,17,23^, CM is usually grouped with other types of immunosuppression. The reported rates of complicated diverticulitis in immunosuppressed patients varies widely, ranging from 30% to 85%^13,23,24^. This is in line with the results of the present study, in which the rate of complicated diverticulitis varied from 39% among patients using other IMs to 90% in patients using > 40 mg of prednisolone equivalent/day. The increased rate of complicated diverticulitis among patients on immunosuppressive medication is largely due to their older age and co-morbidities (outside of immunosuppressive medication). After matching for age and co-morbidities, IM other than corticosteroids interestingly did not increase the rate of complicated diverticulitis. CM has been associated with perforation in diverticulitis (odds ratio 2.7–7.8)^16,25^, and with a higher mortality rate (hazard ratio 1.96)^18^ in diverticulitis. However, it has been unclear whether the increase in mortality is explained by the increase in diverticulitis severity (stage) or by another mechanism that increases mortality regardless of diverticulitis stage. In the present study, there were no significant differences in mortality, length of hospital stay, or the need for surgical intervention/intensive care when CM patients were compared to matched CM-controls with the same diverticulitis stage. It seems that the increased mortality rate is explained by the increased rate of higher initial stage of diverticulitis among patients on CM. Patients whose diverticulitis presented initially as uncomplicated tended to recover well and the progression of diverticulitis into a complicated form was rare even for patients on corticosteroid or other immunosuppressive medication. Other centres have reported similar results: He *et al*.^24^ reported a 93% success rate for conservative treatment in uncomplicated diverticulitis in immunocompromised patients and Ahmadi *et al*.^26^ found no difference in the need for surgical intervention between immunosuppressed and immunocompetent patients presenting initially with uncomplicated diverticulitis. The only stage-specific difference in treatment outcomes was a longer hospital stay for both CM and other IM users compared with their respective control groups. This difference may partly reflect intravenous antibiotic treatment started for most patients with immunosuppressive medication. In the study of Sancho-Muriel *et al*.^27^, treatment results for conservative treatment were similar between severely immunocompromised and immunocompetent patients with diverticulitis complicated with an abscess. This is in line with the findings of the present study, namely that the treatment results within a certain diverticulitis stage did not differ between CM or other IM users and their respective controls.

The mechanisms behind the association between diverticulitis severity and corticosteroid (and its dose) are currently unclear. CM may alter the symptoms of lower stages of diverticulitis leading to selection bias, when only patients with more severe disease seek medical attention. Another possibility is that there is a specific mechanism for CM that increases the risk of bowel perforation in diverticulitis. Possible candidates for this are the glucocorticoid-induced tumour necrosis factor receptor and increased matrix metalloproteinase-9 expression^28^. The increased risk for gastrointestinal perforation among corticosteroid users expands beyond patients with diverticulitis. Corticosteroid increases the risk of intra-abdominal abscess in Crohn’s disease^29^, perforation in peptic ulcer disease^30^, and the risk of complicated appendicitis^31^. CM has also been associated with a higher risk of anastomotic leakage after colorectal surgery^32,33^.

This study has limitations. As a single-centre cohort, the generalizability of the results are limited. The retrospective study design means that the study is prone to selection bias and only associations, not causation, can be reported. PS matching was used to control for confounding factors, but all confounding variables could not be controlled for (for example, CTD, which is tightly linked to corticosteroid use) and residual confounding may remain. Thus, the associations reported here for CM may be due to the underlying disease the medication is used for. However, this limitation was mitigated by conducting sensitivity subgroup analyses by excluding a disease group, which showed similar results to the main analyses. It is not possible to completely separate medication from the underlying disease it is used for. This is particularly so for patients receiving high-dose CM who have a severe disease that requires high-dose treatment. Although corticosteroid use was not associated with worse outcomes within different diverticulitis stages in the present study, these results need to be interpreted with caution because corticosteroid usage may have affected the treatment choices. The stage-specific subgroup analyses led to relatively small subgroups, and a significantly larger patient population may enable differences in treatment results to be found that this study was unable to show. The patient cohort of this study extends from 2006 to 2017; this limits the generalizability of the results because of changes in treatment protocols during and after this period. The focus of this study was the more severe diverticulitis episodes. Many mild episodes of diverticulitis are missing from the data due to the inclusion requirement of diagnosis confirmation by either CT imaging or intraoperative findings. Patients in IM group represent a heterogeneous population with various immunosuppressive drugs. The subgroups for different drug categories were too small to allow comparison between different types of drugs. Sixty-three patients were simultaneously on CM and other IM. This confounding was mitigated by matching CM and CM-control groups for other immunosuppressants and the IM and IM-control groups for corticosteroids, and this overlapping drug use was well balanced in the cohorts. This study is episode-based and a proportion of patients had more than one episode during the study period. Possible confounding was mitigated by conducting a sensitivity analysis where only one (chronologically first) episode was considered in the matched cohorts, with similar results to the main analyses. Because the study includes multiple comparisons, the risk of a type 1 error is increased and therefore the reported *P* values should be interpreted with appropriate caution.

## Supplementary Material

zrag198_Supplementary_Data

## Data Availability

Original study data are not publicly available.
