# Supplementary material for: Association of corticosteroids and other immunosuppressive medications with the severity and outcomes of diverticulitis: propensity score-matched retrospective study
Source: BJS Open. 2026 Nov 13;10(6):zrag198. doi: 10.1093/bjsopen/zrag198 (PMC13767577; doi:10.1093/bjsopen/zrag198)
Supplement: Supplementary file 1 [file zrag198_supplementary_data.docx]

Association of corticosteroids and other immunosuppressive medications with severity and outcomes of diverticulitis: propensity-score matched retrospective study

Johannes M Salusjärvi¹ MD, Laura Koskenvuo¹ MD, PhD, Juha P Mali¹ MD, PhD, Akseli Bonsdorff2 MD, PhD, Ari K Leppäniemi¹ MD, PhD, Panu J Mentula¹ MD, PhD, Ville J Sallinen¹,3 MD, PhD

^1^ Gastroenterological Surgery, Helsinki University Hospital and University of Helsinki, Helsinki, Finland

^2^ University of Helsinki, Helsinki, Finland

^3^ Transplantation and Liver Surgery, Helsinki University Hospital and University of Helsinki, Helsinki, Finland

**Corresponding author:**

**Ville Sallinen**

**Gastroenterological Surgery / Transplantation and Liver Surgery**

**Helsinki University Hospital and University of Helsinki**

**Haartmaninkatu 4**

**00029 Helsinki, Finland**

**Twitter: @villesallinen**

**Email:** [**ville.sallinen@helsinki.fi**](mailto:ville.sallinen@helsinki.fi)

**Supplementary Materials - Index**

| **Supplementary Figures and Tables** |  |
| --- | --- |
| **Supplementary Table S1** | *page 2* |
| **Supplementary Table S2** | *page 2* |
| **Supplementary Table S3** | *page 3* |
| **Supplementary Table S4** | *page 3* |
| **Supplementary Table S5** | *page 4* |
| **Supplementary Table S6** | *page 4* |
| **Supplementary Table S7** | *page 5* |
| **Supplementary Figure S1** | *page 6* |
|  |  |

**Supplementary Figures and Tables**

| Time period | Helsinki 2.0 staging | | | | | Obstruction/fistula | Total |
| --- | --- | --- | --- | --- | --- | --- | --- |
|  | 1 | 2 | 3 | 4 | 5 |  |  |
| 2006-2011 | 632 (66.0 %) | 100 (10.4 %) | 95 (9.9 %) | 67 (7.0 %) | 45 (4.7 %) | 18 (1.9 %) | 957 (100 %) |
| 2012-2017 | 1788 (79.0 %) | 214 (9.5 %) | 118 (5.2 %) | 73 (3.2 %) | 59 (2.6 %) | 10 (0.4 %) | 2262 (100 %) |

**Supplementary Table S1**. Patients divided into two equal time periods

**Supplementary Table S2**. Standardized mean difference for propensity score variables in CM and CM-control groups.

|  | CM | CM-control | SMD |
| --- | --- | --- | --- |
| Number of patients | 179 | 179 |  |
| Age, years, median [IQR] | 70.5 [62.7-77.8] | 72.6 [64.1-82.7] | 0.158 |
| Female sex, n | 133 (74.3 %) | 128 (71.5 %) | 0.050 |
| CKD, n | 11 (6.1 %) | 8 (4.5 %) | 0.075 |
| Cytotoxic medication or ongoing chemotherapy, n | 13 (7.3 %) | 9 (5.0 %) | 0.093 |
| COPD, n | 16 (8.9 %) | 18 (10.1 %) | 0.038 |
| Asthma, n | 47 (26.3 %) | 43 (24.0 %) | 0.051 |
| History of diverticulitis, n | 42 (23.5 %) | 40 (22.3 %) | 0.027 |
| Diabetes, n | 34 (19.0 %) | 38 (21.2 %) | 0.056 |
| Cardiovascular or cerebrovascular disease, n | 39 (21.8 %) | 41 (22.9 %) | 0.013 |
| Dementia, n | 8 (4.5 %) | 9 (5.0 %) | 0.026 |
| Need for assistance prior to admission, n | 31 (17.3 %) | 32 (17.9 %) | 0.015 |
| Liver disease, n | 0 | 1 (0.6 %) | 0.106 |
| Immunosuppressive medication (other than corticosteroid), n | 54 (30.2 %) | 47 (26.3 %) | 0.087 |

CM = corticosteroid medication

SMD = standardized mean difference

SD = standard deviation

CKD = chronic kidney disease

COPD = chronic obstructive pulmonary disease

**Supplementary Table S3**. Standardized mean difference for propensity score variables in IM and IM-control groups.

|  | IM | IM-control | SMD |
| --- | --- | --- | --- |
| Number of patients | 121 | 242 |  |
| Age, years, median [IQR] | 65.6 [58.0-74.0] | 69.0 [57.9-77.6] | 0.134 |
| Female sex, n | 92 (76.0 %) | 184 (76.0 %) | <0.001 |
| CKD, n | 13 (10.7 %) | 16 (6.6 %) | 0.147 |
| Cytotoxic medication or ongoing chemotherapy, n | 3 (2.5 %) | 9 (3.7 %) | 0.071 |
| COPD, n | 4 (3.3 %) | 13 (5.4 %) | 0.101 |
| Asthma, n | 22 (18.2 %) | 52 (21.5 %) | 0.083 |
| History of diverticulitis, n | 38 (31.4 %) | 62 (25.6 %) | 0.128 |
| Diabetes, n | 17 (14.0 %) | 41 (16.9 %) | 0.080 |
| Cardiovascular or cerebrovascular disease, n | 24 (19.8 %) | 39 (16.1 %) | 0.097 |
| Dementia, n | 2 (1.7 %) | 6 (2.5 %) | 0.058 |
| Need for assistance prior to admission, n | 9 (7.4 %) | 21 (8.7 %) | 0.045 |
| Liver disease, n | 2 (1.7 %) | 3 (1.2 %) | 0.035 |
| Corticosterid medication, n | 63 (52.1 %) | 121 (50.0 %) | 0.041 |

IM = immunosuppressive medication (other than corticosteroid)

SMD = standardized mean difference

SD = standard deviation

CKD = chronic kidney disease

COPD = chronic obstructive pulmonary disease

**Supplementary Table S4**. Indication and median dose of corticosteroid medication in the matched CM group

| **Indication for corticosteroid medication** | **n (%)** | **Median daily dose (mg) as prednisolone equivalent [IQR]** | |
| --- | --- | --- | --- |
| Connective tissue disease | 85 (47.5 %) | 5 | [5-10] |
| Tumor/malignancy | 40 (22.3 %) | 60 | [38.5-89.3] |
| Asthma/COPD | 14 (7.8 %) | 10 | [8.8-20] |
| Vasculitis | 10 (5.6 %) | 6.3 | [4.4-42.5] |
| Organ transplant | 3 (1.7 %) | 15 | [2.5-15] |
| Other | 27 (15.1 %) | 16.3 | [5-25] |

IQR = interquartile range

COPD = chronic obstructive pulmonary disease

**Supplementary Table S5**. Indication of immunosuppressive medication (other than corticosteroids) in the matched IM group.

| **Indication for immunosuppressive medication** | **n (%)** | **Most common medication n (%)** | |
| --- | --- | --- | --- |
| Connective tissue disease | 78 (64.5 %) | methotrexate | 53 (67.9 %) |
| Organ transplant | 16 (13.2 %) | tacrolimus | 8 (50 %) |
| Other | 16 (13.2 %) | azathioprine | 6 (37.5 %) |
| Vasculitis | 7 (5.8 %) | methotrexate | 5 (71.4 %) |
| Tumor/malignancy | 4 (3.3 %) | other | 2 (50 %) |

**Supplementary Table S6**. Severity of diverticulitis according to daily corticosteroid dose (predinsolone equivalent) in the matched cohort.

|  |  | | Helsinki stage 2.0 | | | | |  | p^ |
| --- | --- | --- | --- | --- | --- | --- | --- | --- | --- |
|  |  | | 1 | 2 | 3 | 4 | 5 | Total |  |
| CM-control | |  | 105 | 14 | 23 | 15 | 19 | 176 | < 0.001 |
|  | |  | 59.7 % | 8.0 % | 13.1 % | 8.5 % | 10.8 % | 100 % |  |
| CM, patients grouped by daily corticosteroid dose | | 1-5 mg | 31 | 12 | 11 | 5 | 4 | 63 |  |
|  |  |  | 49.2 % | 19.0 % | 17.5 % | 7.9 % | 6.3 % | 100 % |  |
|  |  | 6-10 mg | 14 | 4 | 6 | 5 | 4 | 33 |  |
|  |  |  | 42.4 % | 12.1 % | 18.2 % | 15.2 % | 12.1 % | 100 % |  |
|  |  | 11-20 mg | 6 | 2 | 7 | 5 | 8 | 28 |  |
|  |  |  | 21.4 % | 7.1 % | 25.0 % | 17.9 % | 28.6 % | 100 % |  |
|  |  | 21-40 mg | 2 | 0 | 3 | 7 | 6 | 18 |  |
|  |  |  | 11.1 % | 0 % | 16.7 % | 38.9 % | 33.3 % | 100 % |  |
|  |  | > 40mg | 3 | 0 | 6 | 7 | 15 | 31 |  |
|  |  |  | 9.7 % | 0 % | 19.4 % | 22.6 % | 48.4 % | 100 % |  |
| Total | | | 161 | 35 | 56 | 44 | 56 | 349* |  |
|  |  |  | 46.1 % | 9.2 % | 16.0 % | 12.6 % | 16.0 % | 100 % |  |

Helsinki 2.0 staging: Stage 1, uncomplicated diverticulitis (pericolic gas allowed); Stage 2, complicated diverticulitis with small abscess (<5 cm); Stage 3, complicated diverticulitis with large abscess (≥5 cm) or distant intraperitoneal or retroperitoneal air; Stage 4, Generalized peritonitis without organ dysfunction; Stage 5, generalized peritonitis with organ dysfunction, or fecal peritonitis intraoperatively.

^Pearson Chi-Square test for independence

*missing 9 (6 bowel obstruction, stage NA, 3 corticosteroid dose unclear)

**Supplementary Table S7**. Stage specific outcomes for patients on corticosteroid medication and patients on other immunosuppressive medication with respective propensity score matched control groups.

|  | Modified Hinchey^ | CM | CM-control | p* | IM | IM-control | | p* |
| --- | --- | --- | --- | --- | --- | --- | --- | --- |
| 30-day mortality, n | 1a | 2 (3.0 %) | 1(0.9 %) | 0.656 | 0 (0 %) | | 3 (2.1 %) | 0.515 |
|  | 1b | 3 (11.5 %) | 0 (0 %) | 0.376 | 0 (0 %) | | 3 (13.6 %) | 0.380 |
|  | 2 | 2 (12.5 %) | 1 (7.7 %) | 1.000 | 2 (33.3 %) | | 2 (10.5 %) | 0.490 |
|  | 3 | 8 (20.0 %) | 6 (24.0 %) | 0.943 | 1 (5.6 %) | | 6 (18.2 %) | 0.409 |
|  | 4 | 12 (48.0 %) | 2 (28.6 %) | 0.628 | 2 (50.0 %) | | 10 (45.5 %) | 1.000 |
| Length of stay, days, median, [IQR] | 1a | 2 [1-6] | 1 [0-2] | 0.001 | 1 [0-3] | | 0 [0-2] | 0.050 |
|  | 1b | 5 [4-9] | 3.5 [2-7] | 0.084 | 5 [3-8] | | 5 [4-11] | 0.472 |
|  | 2 | 10.5 [7-28.5] | 11 [9-21] | 0.559 | 15 [8-33] | | 10 [6-24] | 0.514 |
|  | 3 | 11 [8-14] | 8 [5-13] | 0.226 | 12.5 [8-19] | | 10 [8-13] | 0.251 |
|  | 4 | 13 [7-18] | 13 [7-21] | 0.789 | 14 [7-21] | | 11.5[7-18] | 0.758 |
| Surgical intervention during treatment period, n | 1a | 3 (4.5 %) | 4 (3.6 %) | 1.000 | 2 (2.7 %) | | 2 (2.1 %) | 1.000 |
|  | 1b | 5 (19.2 %) | 3 (16.7 %) | 1.000 | 1 (6.7 %) | | 6 (27.3 %) | 0.253 |
|  | 2 | 9 (56.3 %) | 11 (84.6 %) | 0.216 | 5 (83.3 %) | | 8 (42.1 %) | 0.196 |
|  | 3 | 40 (100 %) | 25 (100 %) |  | 18 (100 %) | | 33 (100 %) |  |
|  | 4 | 25 (100 %) | 7 (100 %) |  | 4 (100 %) | | 22 (100 %) |  |
| Need for ICU during treatment period, n | 1a | 1 (1.5 %) | 0 (0 %) | 0.798 | 1 (1.3 %) | | 1 (0.7 %) | 1.000 |
|  | 1b | 1 (3.8 %) | 0 (0 %) | 1.000 | 0 (0 %) | | 1 (4.5 %) | 1.000 |
|  | 2 | 4 (25.0 %) | 2 (15.4 %) | 0.861 | 3 (50.0 %) | | 3 (15.8 %) | 0.245 |
|  | 3 | 12 (30.0 %) | 4 (16.0 %) | 0.328 | 7 (38.9 %) | | 8 (24.2 %) | 0.438 |
|  | 4 | 6 (24.0 %) | 3 (42.9 %) | 0.613 | 2 (50.0 %) | | 7 (31.8 %) | 0.895 |
| ICU-free days within 30d, mean [SD] | 1a | 30 [2] | 30 [2] | 0.124 | 30 [0] | | 30 [2] | 0.486 |
|  | 1b | 28 [5] | 30 [0] | 0.140 | 30 [0] | | 28 [6] | 0.491 |
|  | 2 | 27 [7] | 28 [5] | 0.531 | 21 [13] | | 29 [4] | 0.221 |
|  | 3 | 24 [10] | 24 [10] | 0.555 | 26 [7] | | 25 [9] | 0.766 |
|  | 4 | 20 [12] | 25 [11] | 0.474 | 20 [13] | | 19 [12] | 0.918 |

CM = patients on corticosteroid medication

IM = patients on immunosuppressive medication (other than corticosteroids)

IQR = interquartile range

ICU = intensive care unit

SD = standard deviation

Modified Hinchey classification: 1a, phlegmon, 1b pericolic abscess, 2 pelvic abscess, 3 purulent peritonitis, 4 fecal peritonitis.

^10 patients in corticosteroid cohort and 6 patients in other immunosuppressive medication cohort stage not applicable (bowel obstruction or unclassified peritonitis)
*Pearson chi-square for categorical variables (continuity correction for 2x2 variables) and Mann-Whitney U-test for continuous variables

**Supplementary Figure S1**. Directed acyclic graph of variables assumed to be associated with higher stage of diverticulitis and outcomes
